# Persistent expression of neutrophil gelatinase‐associated lipocalin and M2 macrophage markers and chronic fibrosis after acute kidney injury

**DOI:** 10.14814/phy2.13707

**Published:** 2018-05-23

**Authors:** Hisako Saito, Tetsuhiro Tanaka, Shinji Tanaka, Yoshiki Higashijima, Junna Yamaguchi, Mai Sugahara, Marie Ito, Lisa Uchida, Sho Hasegawa, Takeshi Wakashima, Kenji Fukui, Masaomi Nangaku

**Affiliations:** ^1^ Division of Nephrology and Endocrinology the University of Tokyo Graduate School of Medicine Tokyo Japan

**Keywords:** AKI biomarker, fibrosis, ischemia–reperfusion injury, macrophage polarity

## Abstract

Recent epidemiologic studies revealed a correlation between acute kidney injury (AKI) episodes and the progression to chronic kidney disease (CKD). Although the severity and duration of the initial insult likely predict the development of CKD, information regarding tissue markers predictive of early development of renal fibrosis is limited. We investigated key markers in fibrotic kidney in rats and mice. Seven‐ to eight‐week‐old male Sprague–Dawley rats underwent bilateral ischemia–reperfusion injury (IRI). Kidney tissues were collected to determine the markers correlated with the severity of kidney fibrosis. In a separate set, a specific chemokine (C‐C motif) receptor 2 (CCR2) inhibitor, RS‐102895, was administered to 9‐week‐old male C57BL/6J mice that underwent unilateral IRI (9.2 mg/kg/day in drinking water for 17 days) to investigate whether blockade of the monocyte chemotactic protein‐1 (MCP‐1) signaling was sufficient to prevent fibrosis. Among candidate tissue markers, neutrophil gelatinase‐associated lipocalin (NGAL) and MCP‐1 mRNA expressions were correlated with kidney fibrosis. Studies on macrophage polarity showed that mRNA expression of M2, but not M1 macrophage markers, were correlated with acute‐phase serum creatinine and fibrosis. Pharmacological blockade of the MCP‐1–CCR2 signaling downregulated CCR2, which was insufficient to improve fibrosis in mouse unilateral IRI model, suggesting that additional, redundant pathways contribute to fibrosis. These findings suggested that tissue NGAL expression and M2 macrophage markers are promising markers that show severity of kidney fibrosis. Mechanistic involvement of these markers in CKD pathogenesis warrant additional investigation.

## Introduction

Acute kidney injury (AKI), the rapid loss of kidney function, can be caused by ischemia, exposure to nephrotoxic regents, and urinary tract obstruction. The Kidney Disease: Improvement Global Outcomes (KDIGO) defined AKI as either an abrupt increase in serum creatinine or a decrease in urine output within 48 h (KDIGO Group 2012). AKI remains a significant clinical problem because of the high mortality and morbidity rates (Chertow et al. 2005; Xue et al. 2006; Coca et al. 2009). Kidney function has been speculated to return to normal in survivors of AKI. However, recent studies have shown that a significant proportion of patients with AKI develop a predisposition toward the progression of chronic kidney disease (CKD) (Coca et al. 2009; Lo et al. 2009; Chawla et al. 2011). In contrast, transition mechanisms from AKI to CKD are incompletely understood. To date, therapies for the prevention of AKI have not been established.

To date, many biomarkers for AKI, such as liver‐type fatty acid‐binding protein (L‐FABP), neutrophil gelatinase‐associated lipocalin (NGAL), kidney injury molecule‐1 (KIM‐1), interleukin‐18 (IL‐18), cystatin C, have been developed and are available in clinical care, including early diagnosis of AKI (Alge and Arthur 2015). However, using these biomarkers to investigate the severity of kidney fibrosis is difficult still.

Renal ischemia–reperfusion injury (IRI) is a leading cause of AKI in both allografts and native kidneys (Star 1998). The pathophysiology of renal IRI is complicated because of the simultaneous occurrence of endothelial cell damage, tubular necrosis, tubular apoptosis, inflammation, and tubular cell proliferation, and the difficulty in predicting which patients are likely to develop CKD in the long term.

In this study, we created an AKI to CKD transition model in rats with bilateral IRI and investigated which biomarkers were available to represent kidney fibrosis severity. Of the candidate markers capable of prediction, we focused on the pathogenic role of the monocyte chemotactic protein‐1 (MCP‐1)–chemokine (C‐C motif) receptor 2 (CCR2) signaling, using a specific inhibitor, RS‐102895, in a mouse model of kidney fibrosis caused by unilateral IRI.

## Materials and Methods

### Animal model

Seven‐ to eight‐week‐old male Sprague–Dawley (SD) rats (CLEA Japan, Inc. Tokyo, Japan) were used in this study. The rats underwent bilateral ischemia–reperfusion injury (IRI) after 1 week of acclimatization; both kidney pedicles were clamped for 40–50 min, followed by reperfusion and anesthetized using pentobarbital (Kyoritsu Seiyaku, Tokyo, Japan) through intraperitoneal injection to vary the severity in acute‐phase serum creatinine and kidney fibrosis (40 min; *n* = 12, 42 min; *n* = 6, 45 min; *n* = 10, 50 min; *n* = 6, total *n* = 34). Blood was obtained by performing tail vein puncture at 0 and 48 h. The rats were killed at the end of week 4 and blood was obtained by performing cardiac puncture, and the kidneys were removed for histological evaluation. The serum creatinine levels were measured at 0 and 48 h and at 28 days. Eight‐week‐old sham‐operated SD rats were anesthetized using pentobarbital through intraperitoneal injection and only underwent laparotomy (*n* = 2).

Nine‐week‐old male C57BL/6J mice (CLEA Japan, Inc. Tokyo, Japan) were also used in this study. The mice underwent unilateral IRI after 1 week of acclimatization; the left kidney pedicle was clamped for 27 min, followed by reperfusion and anesthetized using a combination of midazolam (Sandoz, Tokyo, Japan), butorphanol (Meiji Seika Pharma Co., Ltd, Tokyo, Japan), and medetomidine (Kyoritsu Seiyaku, Tokyo, Japan) through intraperitoneal injection. The mice were killed after 14 days, and blood was obtained by performing cardiac puncture, and the left kidney was removed for histological evaluation, and both kidneys were collected to investigate mRNA expression. RS‐102895 (Merck KGaA, Darmstadt, Germany) was dissolved in dimethyl sulfoxide (Wako, Osaka, Japan) (RS‐102895 group) or water containing the same amount of dimethyl sulfoxide (vehicle group) was administered 3 days preoperatively until 14 days postoperatively. The calculated amount of RS‐102895 was 9.2 mg/kg/day per mouse (vehicle; *n* = 6, RS‐102895; *n* = 6).

The Graduate School of Medicine, the University of Tokyo (P14–157, P16–139) approved all the animal experiments, and the animal experiments were performed in accordance with the guidelines established by the Committee on Ethical Animal Care and Use at the University of Tokyo.

### Serum creatinine measurement

L‐type creatinine kit (Wako, Osaka, Japan) was used to measure the serum creatinine in rats with bilateral IRI thrice: preoperatively, 48 h (acute phase), and 4 weeks postoperatively.

### Histological evaluation

Formalin‐fixed, paraffin‐embedded sections (3 *μ*mol/L) were dewaxed and rehydrated through graded alcohols. Masson trichrome staining was used to determine tubulointerstitial injury. Tissue fibrosis was defined as the accumulation of extracellular matrix shown in blue in Masson trichrome staining under low magnification. All the fields from the cortical and the outer medullary areas were selected to measure the areas of tubulointerstitial fibrosis using ImageJ 1.48v software (National Institutes of Health, MD).

### Immunohistochemistry

Formalin‐fixed paraffin‐embedded tissues were sectioned at 3 *μ*m for immunohistochemistry. The following primary antibodies were used: anti‐macrophage (clone ED‐1) (1/400, Merck KGaA, Darmstadt, Germany), rat anti‐mouse F4/80 (1/400, Bio‐Rad Laboratories, Inc. Hercules, CA), and anti‐polyclonal CD206 (mannose receptor) (1/2000, Abcam, Cambridge, UK) were used to investigate active whole and M2 macrophages.

### Real‐time polymerase chain reaction

RNA was isolated from the kidney cortex using RNAiso Plus (Takara, Shiga, Japan) and reverse transcribed with PrimeScript RT Master Mix (perfect Real Time, Takara, Shiga, Japan). A measure of one‐twentieth cDNA was used as a template for subsequent quantification. Polymerase chain reaction (PCR) was run on real‐time PCR: CFX96 System (Bio‐Rad Laboratories, Inc., Hercules, CA), using THUNDERBIRD SYBR QPS‐201 (Toyobo, Osaka, Japan). Real‐time PCR was used to measure the mRNA levels of AKI biomarkers, such as kidney injury molecule‐1 (KIM‐1), liver‐type fatty acid‐binding protein (L‐FABP), NGAL, MCP‐1, and its receptor CCR2, as well as M1 and M2 macrophage markers, to show the correlation with acute‐phase serum creatinine (48 h after bilateral IRI) and the extent of kidney fibrosis. Relative expression levels were calculated using *β*‐actin mRNA as reference. Table 1 shows the primers for quantification.

**Table 1:** List of primers

	Forward (Sequence 5′→3′)	Reverse (Sequence 5′→3′)
Rat		
*β*‐actin	CTTTCTACAATGAGCTGCGTG	TCATGAGGTAGTCTGTCAGG
KIM‐1	AGAGAGCAGGACACAGGTT	ACCCGTGGTAGTCCCAAACA
L‐FABP	CTGAGGACCTCATCCAGAA	CACCCTCCATCTTAACCAC
NGAL	GATGTTGTTATCCTTGAGGCCC	CACTGACTACGACCAGTTTGCC
MCP‐1	ACTGAAGCCAGCTCTCTCTTCCTC	TTCCTTCTTGGGGTCAGCACAGAC
CCR2	CTTAGACCAGGCCATGCAGGTG	ATGTTGAGCTCACTGGTCTGC
IL‐6	CATTCTGTCTCGAGCCC	GCTGGAAGTCTCTTGCGGAG
iNOS	AGAAGTCCAGCCGCACCACC	AAGGCAGCAGGCACACGCAA
arginase 1	TTCTCTAAGGGACAGCCTCG	GCTGTCATTGGGGACATCCA
CD163	ACTCTGAAGCGACGACAGAC	ATGCCAACCCGAGGATTTCAG
CD206	AGTTTAAGCACTGGCTGGCA	AGGCACATCACTTTCCGAGG
Mouse		
MCP‐1	ACTGAAGCCAGCTCTCTCTTCCTC	TTCCTTCTTGGGGTCAGCACAGAC
CCR2	TCAGCTGCCTGCAAAGACCAG	CATACGGTGTGGTGGCCCCT
TNF‐*α*	CATCTTCTCAAAATTCGAGTGACAA	TGGGAGTAGACAAGGTACAACCC
IL‐6	CCAGTTGCCTTCTTGGGACT	GGTCTGTTGGGAGTGGTATCC
iNOS	CACCTTGGAGTTCACCCAGT	ACCACTCGTACTTGGGATGC
CD86	TCCAAGTTTTTGGGCAATGTC	CCTATGAGTGTGCACTGAGTTAAACA
arginase 1	CARGGGCAACCTGTGTCCTT	TCCTGGTACATCTGGGAACTTTC
CD206	TGGATGGATGGGAGCAAAGT	AATGCCAACCTTCCTTGCAG

KIM‐1 (kidney injury molecule‐1), L‐FABP (liver‐type fatty acid‐binding protein), NGAL (neutrophil gelatinase‐associated lipocalin), MCP‐1 (monocyte chemotactic protein‐1) and its receptor CCR2 (chemokine (C‐C motif) receptor 2), TNF‐*α* (tumor necrosis factor‐*α*), IL‐6 (interleukin‐6), iNOS (inducible nitric oxide synthase), CD86, arginase 1, CD163, and CD206 (mannose receptor) were used.

Tumor necrosis factor‐*α* (TNF‐*α*), interleukin‐6 (IL‐6), inducible nitric oxide synthase (iNOS), and CD86 used as M1 macrophage markers, and arginase 1, CD163, and CD206 (mannose receptor) were used as M2 macrophage markers.

### Statistical analyses

Data were expressed as means ± SEM, except for laboratory data (means ± SD). Student unpaired *t* test or Mann–Whitney *U* test were used to analyze the data for the two groups. Differences with *P* < 0.05 were considered statistically significant. Excel and JMP 13^®^ software (SAS Institute Inc., Cary, NC) were used to perform the analyses. In the correlation analyses, *R* ^2^ values of 0.00–0.19 and 0.20–0.39 with *P* < 0.05 were determined to be very weakly and weakly correlated, respectively.

## Result

### Positive correlation between the acute‐phase serum creatinine and the extent of kidney fibrosis

**Figure 1:**
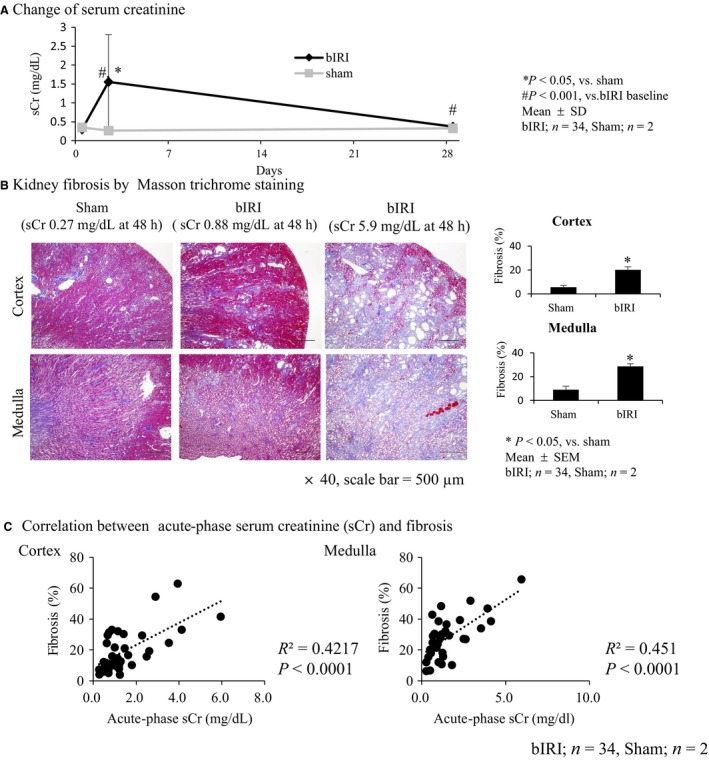
Bilateral IRI (bIRI) was an appropriate model as AKI to CKD transition. (A) Level of serum creatinine (sCr) increased 1.55 ± 0.22 mg/dL 48 h after bilateral IRI (acute phase) compared with the sham group (*P < 0.05), then decreased 0.37 ± 0.07 mg/dL at day 28 (bIRI,* n* = 34; sham, *n* = 2; mean ± SD). (B) Masson trichrome staining of the kidney. bIRI‐induced tubulointerstitial fibrosis compared with the sham group both in the cortex and medulla (*P < 0.05). Tubulointerstitial fibrosis was severe in accordance with acute‐phase sCr (×40, scale bar = 500 *μ*m) (bIRI,* n* = 34; sham, *n* = 2; mean ± SEM). (C) Relationship between acute‐phase sCr and tubulointerstitial fibrosis. A correlation was found between acute‐phase sCr level and the extent of tubulointerstitial fibrosis both in the cortex and medulla (bIRI,* n* = 34; sham, *n* = 2).

The serum creatinine levels 48 h after bilateral IRI (bIRI) were 1.55 ± 0.216 mg/dL (*P* < 0.001, vs. baseline), which remained elevated until day 28 (*P* < 0.001, vs. baseline). In contrast, serum creatinine levels were approximately 0.33–0.36 mg/dL in the sham group during the experiment (Fig. 1A). Bilateral IRI induced kidney fibrosis in the cortex and medulla 28 days after intervention. In contrast, kidney fibrosis did not occur in the sham group (Fig. 1B). The percentage of fibrotic areas in Masson trichrome staining was more severe based on the level of acute‐phase serum creatinine compared with the sham group (Fig. 1B), and they were positively correlated in both the cortex and the medulla (Fig. 1C).

### Correlation between mRNA expression of NGAL and MCP‐1 and acute‐phase serum creatinine and kidney fibrosis

Subsequently, we measured mRNA expression of AKI biomarkers, KIM‐1, L‐FABP, NGAL, and MCP‐1 and its receptor CCR2, in the kidney cortex tissues subjected to bilateral IRI to investigate which AKI biomarkers had a predictive value of kidney fibrosis.

**Figure 2:**
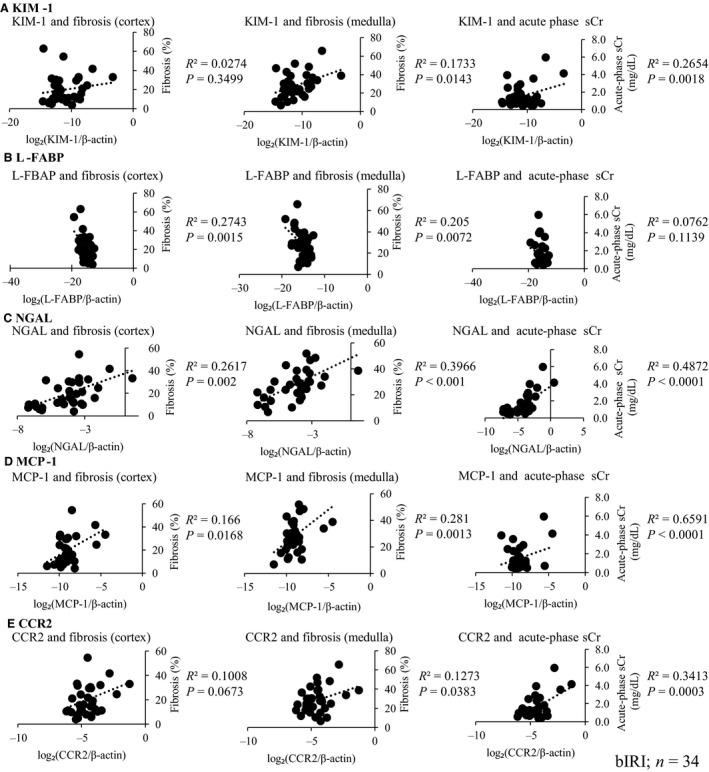
Relationship between mRNA expression of AKI biomarkers and renal fibrosis and acute‐phase serum creatinine (sCr). KIM‐1, L‐FABP, NGAL, MCP‐1, and its receptor CCR2 mRNA were measured. The relative expression levels of NGAL and MCP‐1 mRNA were higher than those of KIM‐1 and L‐FABP at 28 days. KIM‐1 (A) and L‐FABP (B) mRNA expressions were not consistently correlated with all these parameters, but NGAL (C), MCP‐1 (D), and its receptor CCR2 (E) were positively correlated with these parameters, except for the marginal trend between mRNA expression of CCR2 and kidney fibrosis in the cortex (*n* = 34).

Of these markers, the relative expression levels of NGAL and MCP‐1 mRNA were higher than those of KIM‐1 and L‐FABP at 28 days. NGAL, MCP‐1, and CCR2 were positively correlated with kidney fibrosis except for the marginal trend between mRNA expression of CCR2 and kidney fibrosis in the cortex, compared with mRNA expression of KIM‐1 and L‐FABP (Fig. 2A–E).

### Correlation between macrophage infiltration into the kidney and acute‐phase serum creatinine and kidney fibrosis

**Figure 3:**
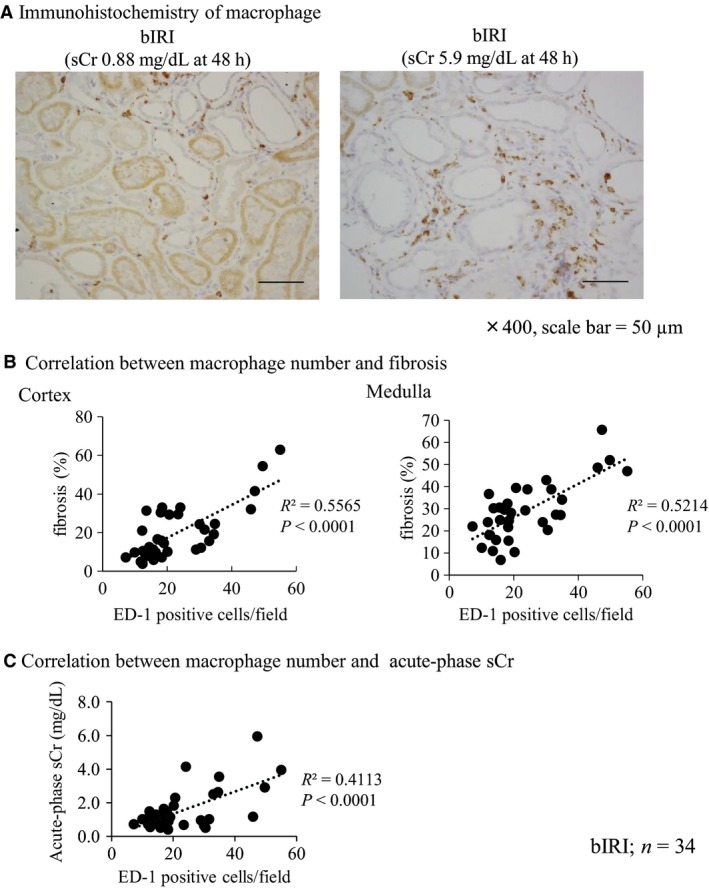
Macrophage infiltration into kidney after IRI was correlated with renal fibrosis and acute‐phase serum creatinine. (A) Immunohistochemistry of macrophage (ED‐1). The number of macrophages increased in proportion to acute‐phase serum creatinine (sCr) level (×400, scale bar = 50 *μ*m). (B) Correlation between the number of macrophages (ED‐1‐positive cells) and renal fibrosis. A positive correlation was found between the number of macrophages and renal fibrosis (bIRI;* n* = 34). (C) Correlation between the number of macrophages (ED‐1‐positive cells) and acute‐phase sCr. A correlation was found between the number of macrophages and acute‐phase sCr (bIRI;* n* = 34).

From the result of the correlation between NGAL and MCP‐1 mRNA expression and acute‐phase serum creatinine and kidney fibrosis, we focused on the relationship of macrophage infiltration in fibrotic kidney. When acute‐phase serum creatinine was high, more severe macrophage infiltration into the kidney was found (Fig. 3A). The number of macrophages was positively correlated with acute‐phase serum creatinine and kidney fibrosis (Fig. 3B and C).

### M2 but not M1 macrophage markers are related to acute‐phase serum creatinine and kidney fibrosis

**Figure 4:**
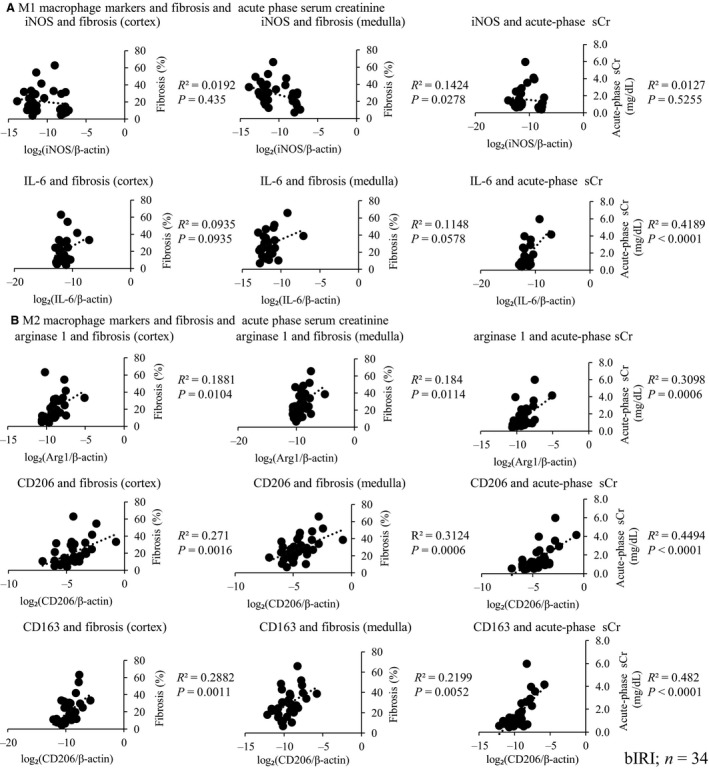
Relationship between M1 and M2 macrophage mRNA expressions and renal fibrosis and acute‐phase serum creatinine (sCr). M1 and M2 macrophage marker mRNA expressions were measured. M1 macrophage mRNA expressions were not correlated with these parameters (A), whereas M2 macrophage mRNA expressions were correlated with these parameters (B) (bIRI;* n* = 34).

We evaluated the relationship between M1 and M2 macrophage markers and acute‐phase serum creatinine and kidney fibrosis. Although the mRNA expression of M1 macrophage markers, IL‐6 and iNOS, was not correlated with acute‐phase serum creatinine and kidney fibrosis, except for the correlation between mRNA expression of iNOS and fibrosis in the medulla and mRNA expression of IL‐6 and acute‐phase serum creatinine (Fig. 4A), the mRNA expression of M2 macrophage markers, arginase 1, CD163, and CD206 (mannose receptor) was significantly correlated with these parameters (Fig. 4B).

**Figure 5:**
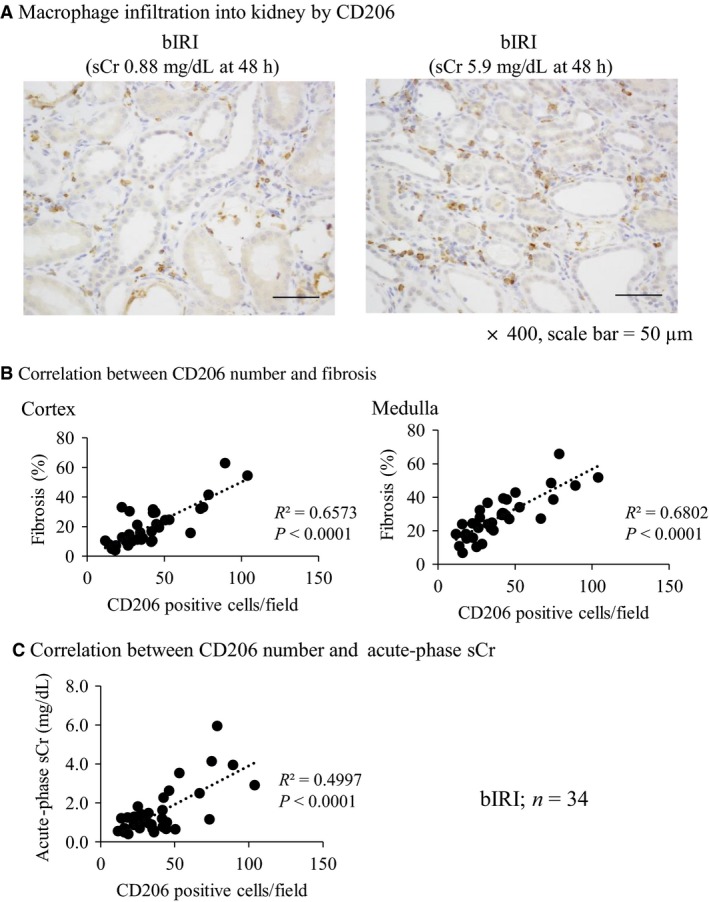
Number of M2 macrophage marker, CD206, is correlated with renal fibrosis and acute‐phase serum creatinine. (A) Immunohistochemistry of M2 macrophage marker, CD206, shows that the macrophage infiltration into the kidney was severe in proportion to acute‐phase serum creatinine (sCr) level (×400, scale bar = 50 *μ*m). (B) A correlation was found between the number of CD206 and renal fibrosis (bIRI;* n* = 34). (C) A correlation was found between the number of CD206 and acute‐phase sCr (bIRI;* n* = 34).

Although quantification of these mRNA markers in the tissues does not accurately represent the presence of M1 and M2 macrophages, it nevertheless suggested the involvement of macrophage skewing. Therefore, we focused on the relationship between M2 macrophage and acute‐phase serum creatinine and kidney fibrosis. If acute‐phase serum creatinine level was high, more severe M2 macrophage infiltration was found (Fig. 5A). The immunohistochemistry of CD206 showed a strong correlation between the number of CD206‐positive cells and both the acute‐phase serum creatinine and kidney fibrosis (Fig. 5B, C).

### CCR2 inhibition reduced mRNA expression of M1 macrophage and inflammatory markers in unilateral IRI model in mice

We hypothesized that macrophage infiltration inhibition into the kidney would decrease kidney fibrosis. Therefore, we used a CCR2 inhibitor, RS‐102895 to focus on macrophage inhibition.

**Figure 6:**
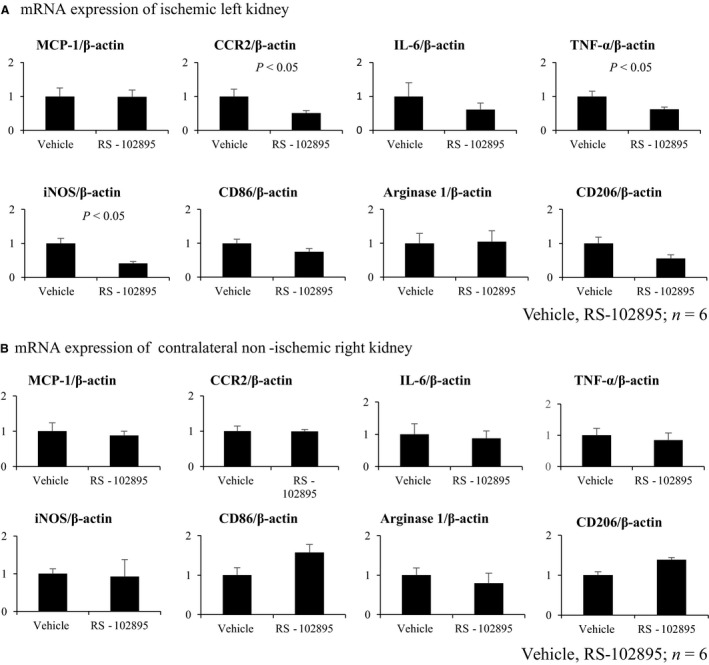
RS‐102895 reduced mRNA expression of CCR2 and M1 macrophage markers, but not M2 macrophage markers in ischemic left kidney. (A) mRNA expression of ischemic kidney. MCP‐1, CCR2, IL‐6, TNF‐*α*, iNOS, CD86, arginase 1, and CD206 mRNA expressions were measured. RS‐102895 reduced mRNA expression of CCR2, M1 macrophage markers, TNF‐*α*, iNOS, but not M2 macrophage markers, arginase 1, and CD206 (vehicle, RS‐102895; *n* = 6). (B) mRNA expression of the non‐ischemic right kidney. MCP‐1, CCR2, IL‐6, TNF‐*α*, iNOS, CD86, arginase 1, and CD206 mRNA expressions were measured in the contralateral non‐clamped right kidney. The expression of these markers was not different between the RS‐102895 and vehicle groups (vehicle, RS‐102895; *n* = 6).

**Figure 7:**
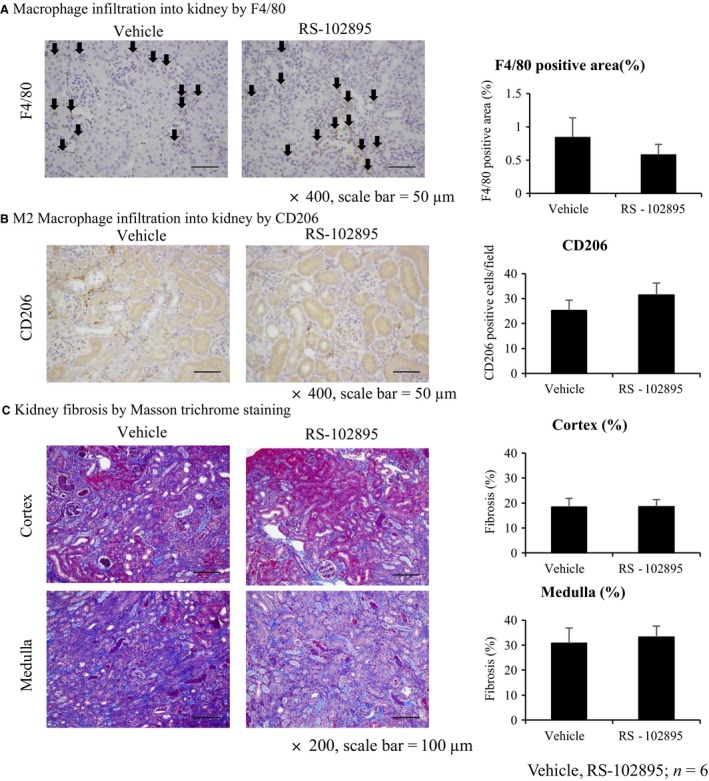
RS‐102895 does not improve renal fibrosis and macrophage infiltration in the unilateral IRI mice model. (A) Immunohistochemistry of F4/80. Macrophage infiltration by immunohistochemistry of F4/80 (arrows) did not reduce by RS‐102895 compared with the vehicle group (×400, scale bar = 50 *μ*m). (B) Immunohistochemistry of CD206. M2 macrophage infiltration did not reduce by RS‐102895 compared with the vehicle group (×400, scale bar = 50 *μ*m). (C) Masson trichrome staining. RS‐102895 did not improve renal fibrosis (×200, scale bar = 100 *μ*m, vehicle, RS‐102895; *n* = 6).

RS‐102895 reduced relative mRNA expression of CCR2 compared with the vehicle group as reported previously, suggesting that the inhibitor blocked the MCP‐1–CCR2 signaling successfully (Kashyap et al. 2016) (Fig. 6A). However, RS‐102895 did not improve whole macrophage infiltration (Fig. 7A), M2 macrophage infiltration (Fig. 7B), and kidney fibrosis (Fig. 7C) compared with the vehicle group. In keeping with these, mRNA expression of M2 macrophage markers, such as arginase‐1 and CD206 did not decrease using RS‐102895, whereas the mRNA expression of M1 macrophage markers, such as iNOS and TNF‐*α*, was reduced (Fig. 6). In contrast, RS‐102895 did not reduce mRNA expression of both M1 and M2 macrophage markers in the contralateral non‐clamped right kidney (Fig. 7B). These results suggested that CCR2 inhibition using RS‐102895 reduced the expression of M1 macrophage markers in the left injured kidney but did not affect M2 macrophage infiltration or improve kidney fibrosis.

## Discussion

Recent studies have confirmed that the likelihood of developing CKD was increased by AKI (Chawla and Kimmel 2012; Coca et al. 2012). The present study was conducted to investigate an optimal tissue marker that could predict the transition to CKD among AKI survivors.

Compared with serum creatinine, many AKI biomarkers are used to detect AKI in the early stages. Some AKI biomarkers, such as NGAL, KIM‐1, and L‐FABP, are reported to be useful in the prediction of CKD after AKI (Malyszko et al. 2008; Ko et al. 2010; Nishida et al. 2010; Zeng et al. 2014; Cooper et al. 2016; Moriya et al. 2017) or CKD progression (Kamijo et al. 2004; Nishida et al. 2010; Moriya et al. 2017). Similarly, MCP‐1 was also reported as an effective AKI biomarker in basic (Munshi et al. 2011) and clinical researches (Shinke et al. 2015; Moledina et al. 2017). Therefore, we chose these four AKI biomarkers as candidates that could predict the progression to CKD after an AKI episode.

Bilateral IRI resulted in the elevation of serum creatinine not only 48 h, but also 28 days postoperatively in this study, suggesting that the capacity of adaptive repair after an AKI episode was overridden by the maladaptive repair with fibrosis (Basile et al. 2016). Ko et al. (2010) reported that progressive kidney fibrosis and concomitant robust transcriptional changes following kidney unilateral IRI in mice sustained during the late repair phase and the regulated genes were largely involved with inflammation and immune responses, which could also participate in progressive kidney injury. KIM‐1 and NGAL showed the highest activity during the experiment, suggesting that these genes might also be useful as surrogate markers in the progression to CKD following IRI and even candidate mediator of the AKI to CKD transition. Nakagawa et al. (2015) also reported that the expression of KIM‐1 and NGAL by microarray analysis correlated with tubulointerstitial fibrosis and tubular cell damage in patients with CKD who underwent biopsy. They concluded that the development of KIM‐1 and NGAL is useful as biomarkers for prediction of kidney injury severity in CKD. These results support the fact that upregulation of mRNA expression of NGAL and MCP‐1, which was not included in their studies, was sustained 28 days after bilateral IRI. Although they did not show the correlation between the expression of these genes and both acute‐phase serum creatinine and kidney fibrosis, our results showed the correlation between NGAL and MCP‐1 mRNA expression and both acute‐phase serum creatinine and tubulointerstitial fibrosis, suggesting that NGAL and MCP‐1 in kidney tissue might not only predict AKI severity but also be good biomarkers to determine kidney fibrosis severity.

NGAL and MCP‐1 are also important inflammatory markers (Forni et al. 2017). Inflammation plays an important role in the pathogenesis of renal IRI (Bonventre and Zuk 2004; Kinsey et al. 2008). Forbes et al. reported that both monocyte/macrophage infiltration and *α*‐SMA positive cells into the kidney interstitium increased after IRI (Forbes et al. 1999). Another animal model of IRI‐induced AKI has identified macrophages as the cell type that accumulates in the kidney in response to tubular cell death and persists with the largest numbers through tubular repair (Lee et al. 2011). In addition, macrophages have infiltrated into the kidney within 24 h after reperfusion in the murine model of IRI (Huen and Cantley 2017). Some groups indicated that the depletion of macrophage before IRI improved kidney injury. In contrast, the depletion of macrophage during the tubular repair phase suppressed kidney recovery (Day et al. 2005; Jo et al. 2006; Vinuesa et al. 2008; Lin et al. 2010; Lee et al. 2011; Ferenbach et al. 2012; Lu et al. 2012; Kulkarni et al. 2014; Clements et al. 2016). These reports suggested that activated macrophages within the kidney promoted tubular injury during the initial phase after IRI, whereas during the late phase, macrophages are required for tubular proliferation during normal repair. However, the mechanisms by which macrophages contribute to kidney fibrosis are not completely understood in sustained or irreversible kidney injury. In addition, our study highlighted macrophages as an important mediator to determine the progression to CKD after AKI, because macrophage infiltration into the kidney was correlated with the acute‐phase serum creatinine level and tubulointerstitial fibrosis.

Macrophages are classified into two phenotypes: M1 (classically activated) and M2 (alternatively activated). M1 macrophages are considered pro‐inflammatory and produce cytokines, such as IL‐1, IL‐6, and TNF‐*α*, whereas M2 macrophages are mainly anti‐inflammatory and pro‐fibrotic and express arginase, CD206 (mannose receptor), IL‐10, and IL‐4 receptor‐*α* (Murray and Wynn 2011; Sica and Mantovani 2012). In this study, the mRNA expression of M1 macrophage markers, iNOS and IL‐6, was not correlated with acute‐phase serum creatinine and tubulointerstitial fibrosis, whereas the mRNA expression of M2 macrophage markers, arginase 1, CD163, and CD206, was strongly correlated with these parameters. In addition, M2 macrophage infiltration into the kidney by immunohistochemistry of CD206 showed a stronger positive correlation between these parameters than that of ED‐1, a pan macrophage marker.

Because mRNA expression of MCP‐1 and its receptor, CCR2, were also correlated with acute‐phase serum creatinine and tubulointerstitial fibrosis in the rat bilateral IRI model, we hypothesized that CCR2 inhibition might improve kidney fibrosis after IRI via reduction of M2 macrophage infiltration.

We performed unilateral IRI in mice administered with a CCR2 inhibitor, RS‐102895, 3 days preoperatively until the end (3 days preoperatively to 14 days). The unilateral IRI model was also confirmed as AKI to CKD transition model (Lai et al. 2014; Lech et al. 2014; le Clef et al. 2016). In our study, we chose 27‐min clamping time and we chose to kill the rats at day 14 because their injured kidney showed severe fibrosis, and their fibrotic lesion was comparable with the extent of the fibrosis in our bilateral IRI model in rats. Indeed, the left kidney with unilateral IRI in mice showed 20–30% tubulointerstitial fibrosis. In addition, kidney fibrosis in the contralateral right kidney did not occur (data not shown). RS‐102895 was administered by drinking water 3 days preoperatively until day 14, in reference to preceding studies demonstrating reduction in the number of interstitial macrophages by RS‐504393 (administered orally twice a day in mouse unilateral IRI model for 4 days (Furuichi et al. 2003) and in mouse unilateral ureteral obstruction (UUO) model for 17 days (3 days preoperatively to 14 days) (Kitagawa et al. 2004). Similarly, Kashyap reported that CCR2 inhibitor, RS‐102895, administered via drinking water 2 days before disease induction until 4 weeks, reduced macrophage infiltration successfully, and improved fibrosis in the renal artery stenosis model (Kashyap et al. 2016). Based on these, we hypothesized that RS‐102895 provided with a similar route and time frame would be sufficient in reducing macrophage infiltration into the kidney and fibrosis. In contrast, RS‐102895 treatment did not reduce tubulointerstitial fibrosis or macrophage infiltration by clamping the kidney, although a decrease in relative mRNA expression of CCR2, as well as an M1 macrophage markers, iNOS and TNF‐*α*, in the RS‐102895 group suggested effective inhibition of the CCR2 signaling by RS‐102895 (Kashyap et al. 2016). This contrasts with a previous study by Furuichi et al., which demonstrated that CCR2 signaling contributed to IRI in the kidney. The degree of interstitial macrophage infiltration was reduced by pharmacological CCR2 inhibition and in CCR2‐deficient mice (Furuichi et al. 2003). This group also reported that blockade of CCR2 improved progressive fibrosis in the UUO model, again using CCR2 knockout mice and CCR2 inhibitors (Kitagawa et al. 2004). The degree of CCR2 inhibition in this study of ischemia–reperfusion, compared with genetic CCR2 knockout, might account for the apparent discrepant findings. However, compared with a previous report (Kashyap et al. 2016), the dosage of RS‐102895 was not small and it is possible that inhibition of the MCP‐1–CCR2 signaling alone was not sufficient to suppress AKI to CKD transition because of many redundant pathways of injury remaining unaffected, and the net continuing injury led to similar degrees of fibrosis. No significant changes in the mRNA expression of M2 macrophage markers, arginase 1, and CD206 might support this view.

The M2 macrophages are important to not only anti‐inflammatory, but also pro‐fibrotic functions, including IRI of the kidney (Kim et al. 2015). Whether the reduction of M2 macrophages in the chronic phase might contribute to the reduction of kidney fibrosis after IRI is an important clinical question. In fact, Moore et al. suggested that mice treated with angiotensin II developed hypertension due to M2 macrophage accumulation in the aortic wall (Moore et al. 2015). Moreover, they showed that treatment of mice using a CCR2 antagonist, INCB3344, even after hypertension has been established, is an effective means of inhibiting aortic macrophage accumulation, preventing vessel fibrosis, and elevated blood pressure. In this study, RS‐102895 did not reduce the accumulation and mRNA expression of M2 macrophage in the injured kidney; hence, further studies are required. However, the use of different experimental settings, such as prolonged RS‐102895 treatment might reduce M2 macrophages and influence renal outcomes, which is conceptually supported by the study by Han et al. (2013), suggesting that the M2 macrophage contributes to kidney fibrosis (Ikezumi et al. 2011; Han et al. 2013).

In summary, some of the AKI biomarkers investigated, such as NGAL and MCP‐1, in tissue were correlated with fibrosis after AKI, in the rat bilateral IRI model. M2, but not M1 macrophage infiltration into the kidney, was significantly correlated with the degree of fibrosis. The pharmacological inhibition of the CCR2 signaling alone by RS‐102895 was not sufficient to prevent the AKI from progressing to CKD in murine unilateral IRI, which could not reduce mRNA expression of the M2 macrophage. Alternatively, the study showed that the suppression of M2 macrophage infiltration in the chronic phase after AKI might potentially be effective to prevent the progression to fibrosis, which requires further investigation.

## Conclusion

The results from the experiments of rat bilateral IRI and mouse unilateral IRI suggested that tissue NGAL and MCP‐1 expression, as well as M2 macrophage markers, are promising markers as screening to determine fibrosis severity after AKI. Mechanistic involvement of these markers in CKD pathogenesis warrant additional investigation.

## Conflict of Interest

HS, TT, ST, YH, JY, MS, MI, LU, SH, TW, and KF declare no relationship with any companies that might have a financial interest in the information contained in this manuscript. MN has honorarium and grants from Astellas, AstraZeneca, Alexion, MSD, Kyowa Hakko Kirin, Daiichi‐Sankyo, Mitsubishi‐Tanabe, Chugai, Kissei, Kureha, Takeda, Boehringer‐Ingelheim, Bayer, Mochida, and JT.
